# Supplementary material for: Empirical methods for controlling false positives and estimating confidence in ChIP-Seq peaks
Source: BMC Bioinformatics. 2008 Dec 5;9:523. doi: 10.1186/1471-2105-9-523 (PMC2628906; doi:10.1186/1471-2105-9-523)
Supplement: Additional File 1 — A variety of html documents from the USeq web site detailing the available applications, their best usage, output file type descriptions, command line menus, etc. [file 1471-2105-9-523-S1.zip › USeqUserGuides/JavaDoc.zip!/JavaDoc/trans/cel/CelLine.html]

CelLine


|  |  |  |
| --- | --- | --- |
| **Overview** \| **Package** \| **Class** \| **Use** \| **Tree** \| **Deprecated** \| **Index** \| **Help** | |  |
| **PREV CLASS**   **NEXT CLASS** | **FRAMES**    **NO FRAMES**     **All Classes** |  |
| SUMMARY: NESTED \| FIELD \| CONSTR \| METHOD | DETAIL: FIELD \| CONSTR \| METHOD |  |


---


## trans.cel Class CelLine

```
java.lang.Object
  trans.cel.CelLine
```

**All Implemented Interfaces:**: java.lang.Comparable

---

``` public class CelLine extends java.lang.Object implements java.lang.Comparable ```

Class for holding info about a text cel file line, enables sorting after rotation.

---

**Constructor Summary**

`CelLine(int x,
int y,
java.lang.String line)`


| **Method Summary** | |
| --- | --- |
| `int` | `compareTo(java.lang.Object obj)`             Sort first by y coordinate, then x. |
| `java.lang.String` | `getLine()` |

**Methods inherited from class java.lang.Object**

`equals, getClass, hashCode, notify, notifyAll, toString, wait, wait, wait`

**Constructor Detail**

### CelLine

```
public CelLine(int x,
               int y,
               java.lang.String line)
```


**Method Detail**

### compareTo

```
public int compareTo(java.lang.Object obj)
```

:   Sort first by y coordinate, then x.

    :   **Specified by:**: `compareTo` in interface `java.lang.Comparable`

---


### getLine

```
public java.lang.String getLine()
```


---


|  |  |  |
| --- | --- | --- |
| **Overview** \| **Package** \| **Class** \| **Use** \| **Tree** \| **Deprecated** \| **Index** \| **Help** | |  |
| **PREV CLASS**   **NEXT CLASS** | **FRAMES**    **NO FRAMES**     **All Classes** |  |
| SUMMARY: NESTED \| FIELD \| CONSTR \| METHOD | DETAIL: FIELD \| CONSTR \| METHOD |  |


---
